# A letter to the editor: the impact of COVID-19 on intercalating and non-clinical medical students in the UK

**DOI:** 10.1080/10872981.2020.1771245

**Published:** 2020-05-23

**Authors:** Mohsin Abedi, Dina Abedi

**Affiliations:** a Faculty of Medical Sciences, Newcastle University, Newcastle-Upon-Tyne, UK; b Barts and the London School of Medicine and Dentistry, London, UK

**Keywords:** Medical education, COVID-19, coronavirus, remote learning, digital health

Dear editor,

Among the many services significantly affected by coronavirus (COVID-19) is the medical education sector – medical students at all stages of medical school have been affected, and the digital transformation of education, which began to take shape prior to the outbreak [1], has undeniably been accelerated.

Medical schools were given the green light to fast-track final year medical students into Interim Foundation Year 1 (FiY1) roles [2]-this has been covered extensively, as has the influx of those returning to work in the NHS. And rightly so. Coverage has also been afforded to those medical students who have had their clinical placements cut short, with innovative new methods such as webinars and various remote, online tools offered to them to delay the somewhat inevitable onset of ‘ring-rust’. However, many intercalating and non-clinical medical students feel forgotten, despite the fact that many are quietly contributing to the strides made by medical educators with regards to remote education delivery.

COVID-19 has left intercalators in limbo – caught between a desire to do their perceived duties as medical students and volunteer in the national effort, and the mandatory workloads they will have been prescribed by their university ahead of upcoming exams or dissertations. While the mobilisation and recruitment of medical students in the wake of this pandemic has been encouraged [3], it is not always possible.

This circumstantial loss-of-role sensation, coupled with the impending imposter-syndrome sure to be felt when they soon return to be in the company of their clinical counterparts, has driven many to contribute in an alternative way.

Junior medical students who are yet to experience clinical placements are in a similar situation, and they too have sought to positively influence the medical world from afar.

Indeed, those not in a clinical setting have been encouraged to take this break in their studies to diversify their skillset and various outputs [4]. Whether that be through involvement in medical technology start-ups disseminating webinars to house-bound students, or by getting involved in research which they would otherwise have struggled to make time for, students are indeed making the most of this opportunity.

So we implore any readers to please spare a thought for the forgotten cohorts. While the rest of the world sped away to deal with COVID-19 at the frontline, the intercalators have been part of a group of individuals providing the backroom support that has kept medical education services running- and the effects of this support will be seen long into the future, as the face of medical education has irreversibly changed.
